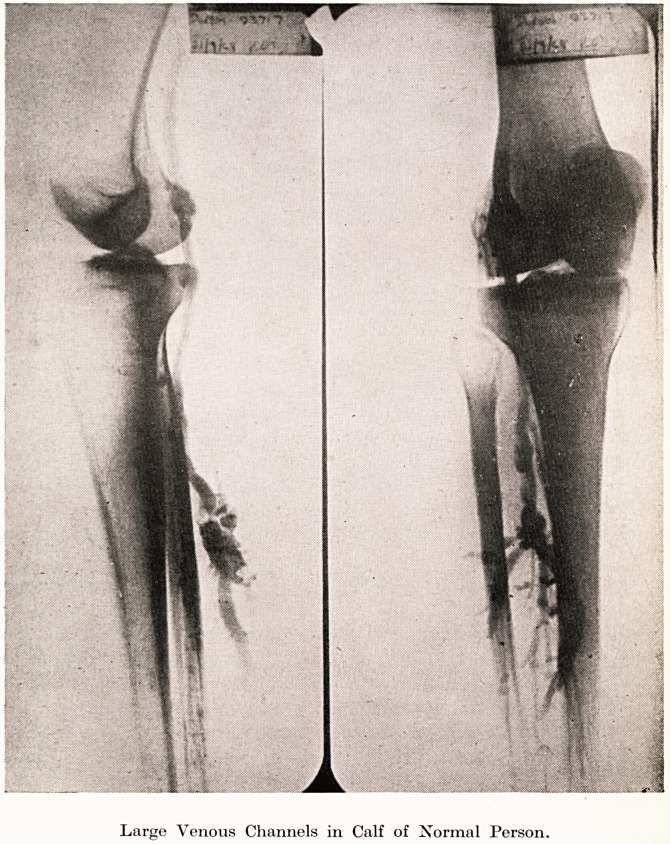# The Surgery of Vascular Disease
*An address delivered before the Bristol Medico-Chirurgical Society, 12th January, 1949.


**Published:** 1949-04

**Authors:** R. Milnes Walker

**Affiliations:** Professor of Surgery, University of Bristol


### THE SURGERY OF VASCULAR DISEASE*

BY

R. Milnes Walker, M.S., F.R.C.S.

*Professor of Surgery, University of Bristol*

---

In this paper I do not propose to discuss the great advances which have been made recently in dealing with the large vessels in the chest and abdomen. Although first reported in 1945, they are all based on the methods of suture which were laid down by Alexis Carrel, as a result of experimental work, more than forty years ago. It was in 1906 that he successfully transplanted the kidneys of one dog into another with suture of their main blood-vessels.[1]

It is more practical to turn our attention to the vascular disturbances in the limbs, for these are common and distressing maladies. We are learning much from angeiography, the method of investigation described in Mr. Moore's paper (p. 37).

### Raynaud's Disease

I am going to confine my remarks to five diseases affecting the peripheral vessels, all but one fairly common. First of all, Raynaud's Disease, described in 1862 in a thesis by Maurice Raynaud on his inauguration as a member of the Academy of Medicine in Paris. The symptoms are too well known for me to reiterate them here. The prognosis is very variable. Some patients appear to get well after a lapse of time and the spasms pass off completely : but the disease may run a course of 5, 10 or even 20 years, although we rarely see it in older people, and one has to bear this in mind in assessing the results of treatment. As we do not know the cause all treatment is really palliative. The avoidance of cold and other medical means may keep the disease in abeyance in the milder cases, but most of the severe ones come to surgical treatment : the usual method is a pre-ganglionic section of the sympathetic nerve fibres of the upper limb, although I am doubtful if this has any advantages over post-ganglionic section except that it avoids Horner's syndrome. The results are not entirely satisfactory. The immediate effects are dramatic—the hands remain pink and warm and, usually, through the first winter this good result is maintained : but some cases relapse during the second or third years after operation, although

*An address delivered before the Bristol Medico-Chirurgical Society, 12th January, 1949.

PLATE VII

Large Venous Channels in Calf of Normal Person.

in most patients the benefit is so marked and lasting that the operation (which carries practically no risks) is well worth while.

THROMBO-ANGEITIS OBLITERANS

I am going to pass on to the disease known as thrombo-angeitis obliterans, first described in detail by Buerger in 1908 and often given his name. It occurs almost exclusively in men between the ages of 25 and 50, and tobacco-smoking seems to play a part in its ætiology ; of 350 patients treated at the Mayo Clinic, only three were non-smokers.[2] The disease usually affects the medium-sized and smaller arteries in the legs, although it may in time involve the vessels of the upper limbs and occasionally the mesenteric, coronary or cerebral arteries. Intermittent claudication is the earliest symptom in 75 per cent. of cases, but ulceration or gangrene may involve the toes or the heel. Usually, the smaller vessels are the worst affected : but since we have taken to making arteriograms of all these patients as a routine we have been surprised to find a number in which the block is a complete obliteration of the popliteal artery, and these patients suffer from severe intermittent claudication as the result of the occlusion of the branches of this artery to the gastrocnemius and soleus muscles. (Plate V, Fig. 1.) If the main arteries lower down are patent, I believe that much can be done by excision of the obstructed portion of the artery and its replacement by a vein graft. We have carried out this procedure in one case, though I regret that subsequently thrombosis took place in the graft.

As a means of testing the value of sympathectomy, various tests can be applied, but injection of the sympathetic ganglion with procaine has the advantage that the patient can take exercise and thus give an indication of its effect on his claudication as well as on his skin-temperature. Sympathectomy, in many cases, staves off the necessity for amputation for a number of years. The prognosis of this disease is not serious as regards life, but a few patients die of coronary, cerebral or mesenteric occlusion. What is in one way more tragic is the risk of the loss of a limb, and in one series it was found that 9 per cent. of patients eventually lost *both* lower limbs. The site of amputation, which usually has to be done for intractable pain, varies from removal of a single digit to an amputation through the thigh, so each case must be judged on its merits with regard to the site of arterial obstruction, and here arteriography may be most valuable.

ARTERIO-SCLEROSIS

In arterio-sclerosis causing gangrene, sympathectomy has little place, and amputation above the knee is usually necessary. However, I would like to draw your attention to gangrene in diabetic patients ; in many instances, this is really an infective gangrene, with all the usual signs of inflammation. Here the treatment is that of an infective lesion, and drastic amputation can be avoided ; while active treatment in controlling the diabetes and chemotherapy are undertaken, abscesses must be widely opened, dead tissue removed, and, if bone is involved, the whole of that bone must be excised ; this may mean the whole of a metatarsal, but even if this is a central one, it is surprising what a useful foot is left when healing takes place.

### Varicose Veins

I cannot omit from this subject the question of varicose veins. The cycle from extensive resection to injection therapy and back to modified excision is almost complete. Nowadays, injection therapy alone is reserved for very localized cases, and advanced cases are treated by ligature and division of the main venous trunks at the groin, the knee and sometimes the ankle as well. It is important to pay attention to the lesser saphenous vein : if its tributaries are affected, it must be divided where it pierces the deep fascia at the level of the crease of the knee. For long it has been taught that, if the deep veins are obstructed, operative treatment or injection of the superficial veins should not be undertaken. I fully agree that injections should not be used in such cases, for they may give rise to thrombosis of some remaining channels by which blood is returning from the leg. But if the main trunk of the internal saphenous vein is incompetent, and blood is flowing downwards instead of upwards, then nothing but good can result from ligature and division of this vein at three or four sites, thus breaking the column of blood which is causing high venous pressure in the foot and lower leg. Contra-indications to active treatment of varicose veins are recent thrombo-phlebitis or the presence of peripheral arterial disease, for in the latter case the congestion of the foot may be beneficial.

While speaking of thrombo-phlebitis, I am going to mention for a moment the question of post-operative thrombosis, which is so apt to start in the deep veins of the muscles of the calf. We have taken the opportunity of studying these veins in normal people when intravenous pyelography is performed, and have been surprised to find the size of these venous channels in persons showing no clinical evidence of varicose veins. (Plate VII.) In these channels, the movement of blood must be very slow and it is no wonder that thrombosis occurs here in a patient confined to bed. The use of the anti-coagulants, heparin or dicoumarol, has revolutionized its treatment, and many cases are up and walking about after only a few days. Occasional thrombosis in a superficial vein, if localized, is best treated by immediate excision, but ligature of the femoral vein for deep thrombosis in order to prevent a release of emboli is rarely called for.

### ARTERIAL EMBOLISM

Finally, a word about arterial embolism ; after hæmorrhage, it is the most urgent surgical emergency, taking precedence over any acute abdominal condition. If the clot is removed within 6 or 8 hrs. complete recovery of the limb is very probable, but if there is longer delay than this the possibility of recovery rapidly diminishes. In my experience all these cases occur in patients suffering from auricular fibrillation, and some of them will die either as a result of their heart-condition, or from further embolism : but an attempt at embolectomy is nearly always worth while, particularly if the obstruction is at the most common site, the bifurcation of the common femoral artery.

During the last Session, we had an excellent exposition of the subject of injury to blood-vessels by Mr. Mason Brown.[3] In civilian life, however, we are faced with very great problems concerning their diseases. There are many points which require further elucidation, and these diseases are, in the main, common ones. Their study needs special technique and special instruments, and I think the problem is sufficiently serious to warrant setting-up in this Region a special unit for the study and treatment of peripheral vascular disease.

### REFERENCES

[1] Carrel, A., quoted by Parkinson, D. and Woodworth, H. C. (1947), *Exper. Med. and Surg*, **5**, 49.

[2] Allen, E. V., Barker, N. W., and Hines, E. A. (1947), *Peripheral Vascular Diseases*, London.

[3] Mason Brown, J. J. (1948), *Bristol Med. Chir. Journ.* **65**, 105.

## Figures and Tables

**Figure f1:**